# Assessing the General Health System of Cyprus: a questionnaire analysis based on public perception

**DOI:** 10.1186/s13690-025-01654-9

**Published:** 2025-07-04

**Authors:** George Evripides, Paul Christodoulides

**Affiliations:** 1 Department of Electrical Engineering, Computer Engineering and Informatics, Cyprus University of Technology, Limassol, Cyprus; 2 Faculty of Engineering and Technology, Cyprus University of Technology, Limassol, Cyprus

**Keywords:** Medical Health System, Healthcare, Regression analysis, Hypothesis test, Analysis of variance

## Abstract

A medical health system (MHS) can be viewed as a diverse network of organizations, experts, and resources devoted to promoting, maintaining, and restoring health and well-being in people and societies. There are numerous MHS throughout the world, and their success depends on a number of aspects that should be met. One such MHS is the newly established General Health System (GHS) of Cyprus, a blend of national HS and Social Health Insurance, supported by beneficiaries, employers, and the state. Following the results of a recent study, based on the same questionnaire, the aim of the present study is to attempt a further assessment on how the various factors (constructs) and demographic variables of people of Cyprus interact in their view of the GHS. The questionnaire was constructed on the basis of the available literature and the information gathered about MHS and their features. It includes 5-point Likert-scale items reflecting relevant factors such as Satisfaction (SAT), Trust (TRU), Reliability (REL), Expectations (EXP), Improvement factors (IMP), and Comparison of the Cyprus Health System before and after the implementation of GHS (COM), and others. The questionnaire was completed by 445 individuals in all districts of Cyprus between January and March of 2024. The descriptive statistics analysis of the questionnaires led to useful findings such as the particularly low mean values for the constructs of SAT, TRU and REL. Another finding is that there is not much variation in the mean values among the categories of groups such as gender, age, education level, annual income, work sector, and residence district. To this end an Analysis of Variance, followed by a multiple regression analysis on all constructs was done, in order to clarify/verify certain issues, which led to conclusions that can be used for the amelioration of the GHS. Demographic variables such as age group and income level have influences on all constructs mentioned above and must be tackled differently by GHS stakeholders. There is a strong correlation between SAT, TRU and REL and COM meaning that if one aims to make reforms on GHS targeting higher SAT, they should enhance TRU, REL, and COM.

**Table:** 

Text box 1. Contributions to literature
• Universal health coverage (UHC) promotes equity and prevents financial hardship. Cyprus’ General Health System (GHS) (since 2019) aims toward UHC, however patient perspectives are missing. This research sheds light on public satisfaction, trust, and reliability of GHS.
• This questionnaire-based study evaluates the GHS and offers stakeholder insights using ANOVA and multiple regression. The study explores how demographics and other factors interact and affect user evaluations and suggests system performance improvements.
• Trust and reliability boost satisfaction, providing evidence-based health-system reforms. The paper promotes evidence-based health policies worldwide by providing a replicable evaluation technique for UHC changing nations.

## Introduction

The medical health system (MHS), among which national health systems (NHS), is a comprehensive network of institutions, professionals, and resources dedicated to promoting, maintaining, and restoring health in individuals and communities. It includes healthcare providers such as physicians, nurses, therapists, and pharmacists, who deliver essential services in hospitals, clinics, and nursing homes [1, 2]. Key components also encompass health insurance, regulatory agencies, medical research, and public health initiatives. A well-functioning MHS ensures access to quality care, contributes to public well-being, and supports socio-economic development. Government oversight and financing mechanisms, including public programs and private insurance, are pivotal for maintaining standards of quality, safety, and equity.

The importance of a robust MHS for individuals cannot be overstated. The main aspects that should be met so that a MHS can be considered as successful include Access to Healthcare Services, Healthcare Quality, Disease Prevention and Health Promotion, Emergency and Trauma Care, Financial Protection, Health Equity and Social Justice, and Economic Development [3–6]. In summary, a well-functioning MHS is essential for safeguarding the health and well-being of individuals, promoting public health, and contributing to social and economic development. It serves as a cornerstone of any society’s efforts to ensure that its population can lead healthy, productive lives. Its overarching goal is to provide comprehensive healthcare services, ranging from prevention and diagnosis to treatment and rehabilitation, to individuals and communities. Healthcare providers deliver essential medical care in diverse settings, supported by healthcare facilities equipped with advanced infrastructure and resources. Financing mechanisms, including public programs and private insurance, ensures access to care for all. Government oversight and regulation maintain standards of quality, safety, and equity within the system.

Public satisfaction with the MHS serves as an indicator utilized by the World Health Organization (WHO) to assess the quality of the healthcare system [7]. Satisfaction with the MHS, while not exhaustive in representing all aspects of MHS quality, constitutes one dimension of that quality. Political leaders and policymakers worldwide are increasingly aware that the stability of the MHS is contingent upon sufficient public satisfaction [8, 9]. Thus, the inquiry is whether particular demographic groups, categorized by education and income, exhibit greater satisfaction in certain countries compared to others as a result of varying levels of financial investment in the healthcare system. The satisfaction of an individual with the MHS may also be influenced by their socio-economic status (SES). Prior studies indicate that socially disadvantaged individuals face challenges in successfully navigating the MHS due to the restricted availability of health-related resources [10]. The MHS can be analyzed using the framework of institutional interactions. Research indicates that health care providers allocate more time to patients when the patients or their companions possess specific medical terminology and pertinent information, facilitating effective communication. This dynamic contributes to disparities in treatment between individuals of lower socioeconomic status (SES) and those of higher SES [11–13].

In a previous paper [6], a first assessment on possible strengths and weaknesses of the newly founded Cyprus General Health System (GHS) was attempted through a construction and completion of a questionnaire (survey performed in 2024). The authors followed certain surveys in MHS that included important relevant factors [14]. GHS combines National Health Service and Social Health Insurance supported by beneficiaries, employers, and the state. Services are provided by public and private firms. Population-wide outpatient healthcare was implemented by GHS in June 2019. A broad overview of some key national MHS [15–18], along with a comparison of MHS frameworks was also addressed. Highlighting their features, strengths, and weaknesses. The authors also addressed facts about MHS in EU, a member of which is Cyprus, presenting number of practicing physicians, number of physicians by specialist, practicing physicians per 100,000 inhabitants, proportion of physicians, by age and gender [19].

Among the conclusions were the low mean values for the constructs of Satisfaction, Trust and Reliability, all well below the median value of 3. On the other hand, the mean value for Expectations was over 4, showing consistency by the respondents who are not satisfied so far and they expect much more by GHS. Another finding is that there is not much variation in the mean values among the demographic categories of groups such as gender, age, district of residence, annual income and work sector. These results came as no surprise, considering the results of older surveys for other MHS. The current study takes over from that study, trying to perform a deeper analysis on the particular relationships and inter-influences among the various factors (questionnaire constructs and demographic variables), based on analysis of variance (ANOVA) and multiple regression.

There is a growing body of literature examining public perception of healthcare systems through questionnaire-based studies [20–22], among which several deal with ANOVA and multiple regression. These studies consistently identify factors such as communication, accessibility, staff professionalism, and system efficiency as key determinants of patient satisfaction. For example, in the United Kingdom, patients prioritize staff empathy and clear communication [21]; in Europe, satisfaction is shaped by access and interpersonal care [23]; and in South Africa, satisfaction is influenced by accessibility and medication availability [24]. Similar patterns have been reported in Morocco [25], Pakistan [26], Japan [27], the United States [28, 29], Turkey [30], Spain [31], China [32, 33], India [34], and in specific models such as the Maryland All-Payer Model [35]. The relevant research in the literature collectively underscores the importance of patient-centered care, emphasizing the necessity for healthcare systems to prioritize both technical and interpersonal aspects of service delivery. A balanced approach integrating patient feedback, operational improvements, and policy adaptations is essential for enhancing patient satisfaction and overall healthcare efficacy.

The primary purpose of this research is to provide an in-depth evaluation of the newly established GHS of Cyprus. Specifically, this study aims to analyze how various factors – such as satisfaction, trust, reliability, expectations, improvement factors, and comparison with the pre-GHS system – interact with demographic characteristics (including gender, age, income, education, work sector, and residence district). Utilizing a structured questionnaire and employing statistical methods like Analysis of Variance (ANOVA) and Multiple Regression, the research seeks to identify key determinants influencing public perception and satisfaction with the GHS. The ultimate goal is to offer actionable insights that could guide stakeholders in refining and enhancing the effectiveness of the GHS. The rest of the paper is organized as follows. In Sect. 2, the methods and materials used for achieving the goals of the current paper are presented, with details on the construction of the questionnaire and data collection also given. In Sect. 3 the statistical analysis of the questionnaire is presented, consisting of the results on Reliability and Correlations’ Analyses, Analysis of Variance and Multiple Regression for several constructs. Finally, a discussion of the results and conclusions are given in Sects. 4 and 5 respectively.

## Methods and materials

The present investigation began by studying already deployed medical/national health systems (MHS/NHS) to determine how they run, how they compare, and what factors regulate them. It helps to remember MHS facts in many nations, especially EU countries like Cyprus, to better grasp the GHS. Due to disparities in healthcare delivery structures, financial mechanisms, cultural contexts, and socio-economic considerations, comparing national MHS internationally is difficult. Due to the complexity and diversity of MHS worldwide, comparing country MHS with numbers and diagrams would be difficult. A general comparison framework includes Healthcare Financing, Coverage and Access, Quality of Care, Healthcare Delivery, Regulatory Framework, Public Health Initiatives, Healthcare Workforce, Equity and Accessibility, Health Information Systems, and Emergency Preparedness [3, 36–38].

It is important to note that obtaining comprehensive and up-to-date data for all countries can be challenging, as MHSs are constantly evolving and data collection methods may vary. Additionally, the interpretation of data can be complex due to differences in population demographics, cultural factors, and healthcare priorities. Therefore, any comparison should be done with caution and with an understanding of the context in which the data was collected. A broad overview of some key national MHS [15–18] can lead to features, strengths, and weaknesses thereof. Studying facts about MHS in EU with regard to number of practicing physicians, number of physicians by specialist, practicing physicians per 100,000 inhabitants, proportion of physicians, by age and gender [19], can set the state of Cyprus in that framework as a member state.

The second step was to offer timeframes and implementation data for the MHS of Cyprus, the study’s subject. Cyprus’s General Healthcare System (Geniko Systima Ygeias, GeSY) is a blend of National Health Service and Social Health Insurance supported by beneficiaries, employers, and the state. Public and contracted private providers provide services. GHS’s initial implementation began in June 2019 and provided population-wide outpatient healthcare. A National Health Insurance Fund managed by the Health Insurance Organization (HIO) covers public and private provider treatments. The State Health Services Organization took over public hospitals from the Ministry of Health to create a new quasi-market, separating purchaser, supplier, and regulator. Family physicians, outpatient specialists, drugs, and laboratories are available for low user fees. The second phase began in June 2020 with integrated hospital care, added specialist pharmaceuticals in September, and ended in December 2020. The reform is a major step toward universal health coverage, reducing out-of-pocket costs and access to care [14]. Appendix 1 describes the GHS reform schedule and qualified beneficiaries.

As the goal here was to conduct a questionnaire for the evaluation of the Cyprus MHS, one could follow certain important factors related to surveys in MHS, namely Assessment of Access, Quality of Care, Identifying Gaps, Health Outcomes, Policy Evaluation, Equity and Inclusivity, as well as Future Planning [14].

Based on literature and the steps above, a questionnaire was created to assess MHS, including Satisfaction (SAT), Trust (TRU), Reliability (REL), Expectations (EXP), Improvement factors (IMP), and Comparison (COM) of the Cyprus Health System before and after GHS implementation. Each variable was represented by categorized Likert-scale questions in the questionnaire. These questions were chosen based on theoretical foundations, a literature review (e.g., [20–22]), and discussions with the authors’ colleagues and other stakeholders. Responses were anticipated to help evaluate system efficacy and identify areas for improvement. Surveys are essential for assessing NHS performance and improving healthcare access, quality, and equity.

Upon the first finalization of the questionnaire by the authors in December 2023, a pilot survey in all districts of Cyprus followed at the beginning of January 2024. The pilot survey included in person meetings with 10 female and 10 male participants – namely 3 doctors, 7 individuals aged 25–55, 5 students and 5 pensioners. Following these personal meetings, several questions (~ 10 of them) were modified to prevent confusion. The final version of the questionnaire (see Appendix 2) consisted of the following sections: (i) Demographic information about the respondent, which included gender, age group, education level, annual income, work sector, residence district; (ii) Likert scale (1–5) questions covering the six factors mentioned above, as well as factors Health Status (HST) and Emotional Intelligence (EQ).

**Fig. 1:**
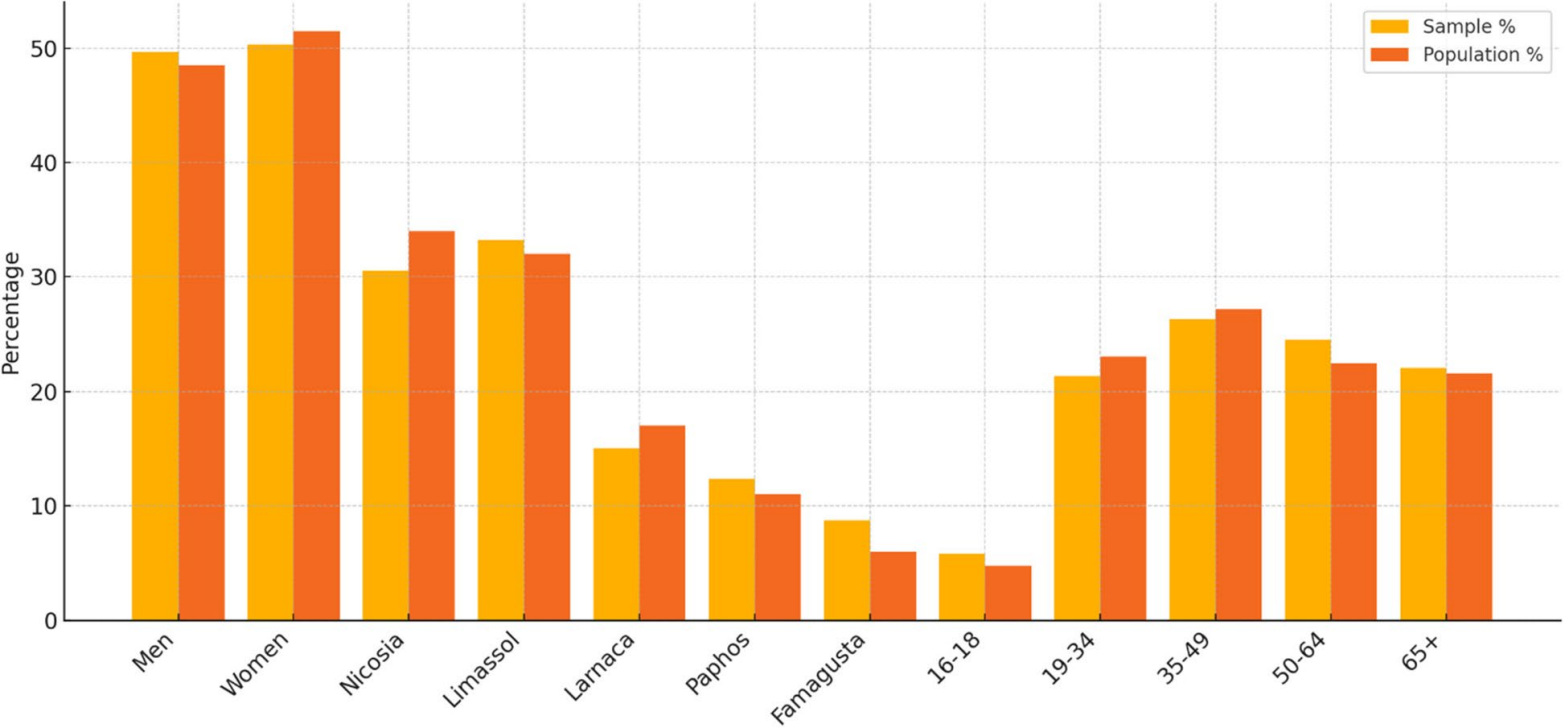
Demographic characteristics – Sample vs Population in the framework of questionnaire analysis for assessing the General Health System of Cyprus (survey performed in 2024)

Data collection, starting in the third week of January 2024, was conducted by skilled enumerators via oral or telephone interviews utilizing tablets, or through the completion of a paper version of the questionnaire by the respondents, which was returned to the enumerators. The collection of 445 completed questionnaires was concluded in mid-March 2024. Furthermore, pursuant to the Law, the Statistical Service is mandated to regard all collected information as secret and to utilize it exclusively for statistical purposes. Regarding how the basic characteristic of the respondents compare with those of total population who utilize the GHS, a considerable effort was made to be in close agreement in regards to gender, age group and District of Residence (see Fig. 1).

Already, in Evripides et al. [6] the descriptive statistical analysis of 445 completed questionnaires was used for the evaluation of GHS. Here the authors continue with a more exhaustive statistical analysis including Spearman’s Correlation Analysis, Analysis of Variance based on demographic characteristics and multiple Regression Analysis, taking into consideration factors like emotional intelligence together with the health status of respondents to act as predictors for the Satisfaction, Trust, Reliability, Expectations, Comparison and Improvement Factors.

## Statistical analysis and results

**Fig. 2:**
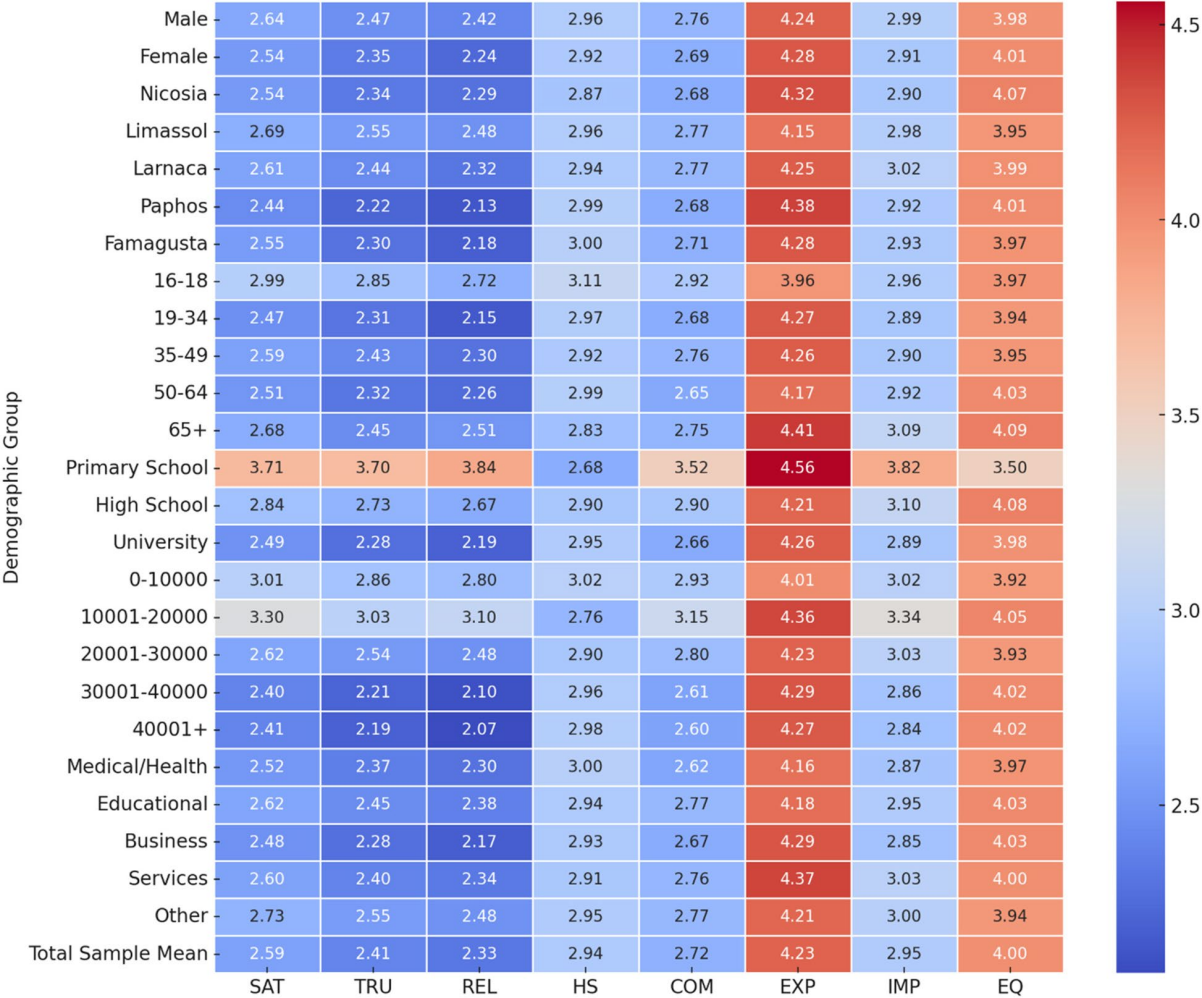
Heatmap of mean values (arising from 5-point Likert scaling) for each construct across demographics (including total sample mean) in the framework of questionnaire analysis for assessing the General Health System of Cyprus (survey performed in 2024). (SAT: Satisfaction, TRU: Trust, REL: Reliability, HST: Health Status, COM: Comparison of the Cyprus Health System before and after the implementation of the General Health System, EXP: Expectations, IMP: Improvement factors, EQ: Emotional Intelligence)

The data obtained from the designed questionnaire were inserted to a MS Excel and subsequently into SPSS, where all analyses were performed. Descriptive statistics was used to summarize and organize the obtained data in a meaningful way. Figure 2 shows results for the mean, based on the 88 questions (items) answered by the 445 participants, which are grouped into 8 constructs, namely SAT, TRU, REL, COM, EXP, IMP, HST and EQ. The mean value for nearly all items and the average of SAT, TRU, REL, COM are well below 3 (the theoretical median), indicating a public having a negative view on GHS. The well above 3 average of EXP shows a public expecting much more from the GHS, verifying the results for factors SAT, TRU, REL and COM. A very interesting point of investigation was whether or not the results differed according to gender, age group, education level, annual income, working sector and residence district. A summary of that analysis is as follows.

- SAT, TRU, REL: Age group 16–18 seems to have a higher value; higher value for Primary Education; higher value for Annual Income less than 20,000 euro.
- HST: Age group 16–18 seems to have a higher value; higher value for Primary Education; higher value for Annual Income less than 20,000 euro.
- COM: Age group 16–18 seems to have a higher value; higher value for Primary Education; higher value for Annual Income between 10,001–20,000 euro.
- EXP: Age group 16–18 seems to have a higher value; higher value for Primary Education.
- IMP: Age group 65+ seems to have a higher value; higher value for Primary Education; higher value for Annual Income between 10,001–20,000 euro.
- EQ: Age groups 50+ seems to have a higher value; lower value for Primary Education; lower value for Annual Income less than 10,000 euro.

Construct reliability was examined through calculation of Cronbach’s alphas, yielding satisfactory results for all constructs, which Cronbach’s alpha values larger than 0.60, namely 0.92 for SAT, 0.93 for TRU, 0.91 for REL, 0.88 for COM, 0.90 for EXP, 0.85 for IMP, 0.67 for HST, and 0.70 for EQ. In addition, correlation Analysis [14, 39, 40] was performed using Spearman’s rank Correlation Coefficients. Table 1 presents a correlation matrix showing the relationships among variables. Correlation r indicates the strength and direction of linear associations between pairs of variables. In general, Strong positive correlation suggests that the constructs may share underlying drivers or dimensions. The values of r(SAT, TRU), r(SAT, REL), and r(TRU, REL) indicate strong positive correlations. Additionally, the values of r(SAT, COM), r(SAT, IMP), r(TRU, COM), r(TRU, IMP), r(REL, COM), r(REL, IMP) and r(COM, IMP) indicate moderate positive correlations. The SAT, TRU and REL, close interrelation highlights their conceptual alignment, reinforcing one another, potentially forming a cohesive construct of overall well-being or social alignment. COM and IMP moderate correlations with SAT, REL and COM, suggest that perceived comparison and improving factors constructs are central factors influencing these constructs.

**Table 1:** Spearman’s correlations between constructs in the framework of questionnaire analysis for assessing the General Health System of Cyprus (survey performed in 2024)

	SAT	TRU	REL	HST	COM	EXP	IMP	EQ
SAT	1.00							
TRU	0.64^a^	1.00						
REL	0.65^a^	0.66^a^	1.00					
HST	0.22^a^	0.05	−0.07	1.00				
COM	0.60^a^	0.60^a^	0.53^a^	0.19^a^	1.00			
EXP	0.09	−0.17^a^	0.01	−0.15^a^	0.19^a^	1.00		
IMP	0.54^a^	0.57^a^	0.59^a^	−0.07	0.56^a^	0.17^a^	1.00	
EQ	−0.27^a^	−0.01	−0.04	−0.47^a^	−0.21^a^	0.16^a^	−0.05	1.00

^a^Correlation is significant at 0.01 level (2-tailed)

*SAT* Satisfaction, *TRU* Trust, *REL* Reliability, *HST* Health Status, *COM* Comparison of the Cyprus Health System before and after the implementation of the General Health System, *EXP* Expectations, *IMP* Improvement factors, *EQ* Emotional Intelligence

The weak negative correlations between EQ and variables such as TRU, REL and IMP are notable, indicating that emotionally intelligent individuals focus less on these constructs or approach them differently. EXP shows generally weak correlations with other variables, particularly SAT and REL; this suggests EXP may operate more independently in the context of this analysis. A similar behavior is observed for HST.

### Analysis of variance

Analysis of Variance (ANOVA) [41, 42] is performed to test the hypothesis that k Normal means are equal. This is done by comparing variance estimates of given observations from different batches (methods, treatments, etc.) called mean sums of squares or simply mean squares. The method is again based on an F Test. There are three important assumptions that should be satisfied for a one-way analysis of variance to be applied. The observations are obtained independently and randomly from the populations defined by the factor levels. The population of each factor level is (approximately) normally distributed. These normal populations have a common variance. Always the null hypothesis is that there is no difference between the population means against the alternative hypothesis that not all population means are equal. At least two differ in the mean values. ANOVA deals with parameters such as: Sum of Squares (SS), which gives the variance explained by between-group differences (numerator) and within-group differences (denominator); degrees of Freedom (df), which refers to the number of groups (between-group) and residuals (within-group); Mean Square (MS), which is SS divided by df; F-statistic, which is the ratio of between-group variance to within-group variance; *p*-value, which is the probability of observing differences due to chance; F_cr_, which is the critical threshold for significance at the chosen confidence level (usually *p* < 0.05). Table 2 shows the results of the performed ANOVA.

**Table 2:** Source of Variation between and within demographic groups – ANOVA in the framework of questionnaire analysis for assessing the General Health System of Cyprus (survey performed in 2024)

**Factor**	**Gender** ^a^F_cr(df=1)_ = 3.86	**Age group** ^a^F_cr(df=4)_ = 2.39	**Education level** ^a^F_cr(df=2)_ = 3.02	**Income level** ^a^F_cr(df=4)_ = 2.39	**Work sector** ^a^F_cr(df=4)_ = 2.39	**Residence district** ^a^F_cr(df=4)_ = 2.39
	F/*p*-value	F/*p*-value	F/*p*-value	F/*p*-value	F/*p*-value	F/*p*-value
SAT	3.54/.061	4.95/.001	25.17/.000	38.31/.000	1.94/.102	2.20/.068
TRU	3.97/.047	4.39/.002	35.55/.000	30.86/.000	1.88/.113	3.80/.005
REL	7.67/.006	6.45/.000	39.77/.000	42.40/.000	2.38/.051	3.69/.006
HST	2.96/.086	9.96/.000	4.26/.015	8.02/.000	1.46/.212	3.32/.011
COM	1.76/.186	2.27/.060	18.32/.000	24.18/.000	1.70/.148	0.80/.526
EXP	0.76/.384	5.91/.000	1.38/.252	3.31/.011	3.13/.015	3.23/.012
IMP	3.73/.054	3.28/.011	45.70/.000	14.42/.000	2.91/.021	1.04/.388
EQ	0.67/.403	3.06/.017	7.50/.001	1.57/.181	0.76/.554	1.89/.111

^a^critical at the .05 level

*SAT* Satisfaction, *TRU* Trust, *REL* Reliability, *HST* Health Status, *COM* Comparison of the Cyprus Health System before and after the implementation of the General Health System, *EXP* Expectations, *IMP* Improvement factors, *EQ* Emotional Intelligence

Based on the table, which examines differences between demographic category groups for each construct, one may conclude that:

- REL shows strong significant differences (*p* < 0.01), TRU shows significant difference (*p* < 0.05), while SAT, HST and IMP show moderate significant difference (*p* < 0.10) among gender groups. No significant difference is found for COM, EXP and EQ.
- SAT, TRU, REL, HST, EXP show strong significant differences (*p* < 0.01), while COM, IMP and EQ show significant difference (*p* < 0.05) among age groups.
- SAT, TRU, REL, COM, IMP, EQ show strong significant differences (*p* < 0.01), while HST shows significant difference (*p* < 0.05) among education level groups. No significant difference is found for EXP.
- SAT, TRU, REL, HST, COM, IMP show strong significant differences (*p* < 0.01), while EXP shows significant difference (*p* < 0.05) among income level groups. No significant difference is found for EQ.
- REL, EXP, IMP show significant differences (*p* < 0.05) among work sector groups. No significant difference is found for SAT, TRU, HST, COM, EQ.
- TRU, REL show strong significant differences (*p* < 0.01), HST, EXP show significant difference (*p* < 0.05), while SAT shows moderate significant difference (*p* < 0.10) among residence district groups. No significant difference is found for COM, IMP and EQ.

Some remarks that may explain the results obtain above are the following:

- Older age groups might have lower HST scores due to natural aging effects, whereas younger groups could perceive better health.
- Younger individuals may have higher EXP due to ambition, while older groups might have tempered EXP based on experience.
- Younger age groups may prioritize IMP more, while older groups might focus on maintaining stability.
- Older individuals might score higher EQ due to greater life experiences and emotional regulation.
- All constructs exhibit significant differences among age groups, underscoring the influence of age as a key factor.
- Education level strongly influences perceptions of opportunities for IMP as well as SAT, TRU, REL.
- Education level may correlate with better HST awareness and access to healthcare resources.
- EQ varies significantly with education, suggesting education level plays a role in EQ development.
- All constructs except EQ exhibit significant differences among income level groups, underscoring the influence of income level as a key factor.
- EQ may not be strongly tied to income level and might be influenced more by other factors like personality or life experiences.
- SAT, TRU, HST, COM, and EQ appear consistent across work sector, indicating that these may be driven by broader organizational or individual factors rather than the specific work environment.
- TRU and REL demonstrates the strongest statistical significance, suggesting substantial differences in perceptions of TRU and REL among residence district, likely driven by disparities in infrastructure, services, or leadership.
- HST also shows significant variation across residence district, with issues such as access to healthcare, environmental conditions, and public health initiatives contributing to these differences.
- EXP also differ significantly by residence district, reflecting regional differences in resources, economic conditions, or cultural expectations.

### Multiple regression analysis

When one wishes to understand the relationship between a single predictor variable and a response variable, they often use simple linear regression. However, if one would like to understand the relationship between multiple predictor variables and a response variable then they could instead use multiple linear regression [43]. If there are p predictor variables, then a multiple linear regression model takes the form:

1 $$Y=\beta_0+\beta_1\;X_1+\beta_2\;X_2+\dots+\beta_\rho\;X_\rho+\varepsilon,$$

where Y is the response variable, X_j_ is the j^th^ predictor variable, β_j_ is the average effect on Y of a one unit increase in X_j_, holding all other predictors fixed, and ε is the error term. The values for β_0_, β_1_, β_2_, …, β_p_ are chosen using the least square method, which minimizes the sum of squared residuals (RSS).

A multiple regression analysis has been performed for each of the six constructs, namely SAT, TRU, REL, COM, EXP, IMP, being the response (dependent) variables, with constructs HST and EQ, the demographic factors Gender, Age, Education, Income, Work, Residence district, as well as the rest of the six above-mentioned constructs, being the predictor (independent) variables. The results are shown in Table 3.

**Table 3:** Multiple regression for seven constructs as independent variables, and six demographic groups and eight constructs as independent variables in the framework of questionnaire analysis for assessing the General Health System of Cyprus (survey performed in 2024)

**Dependent Y/** **Independent X**_**i**_	**SAT** F = 215.81* R^*2*^ =.87	**TRU** F = 203.11* R^*2*^ =.86	**REL** F = 185.42* R^*2*^ =.85	**COM** F = 156.70* R^*2*^ =.83	**EXP** F = 16.15* R^*2*^ =.33	**IMP** F = 33.23* R^*2*^ =.50
	β_i_/*p*-value	β_i_/*p*-value	β_i_/*p*-value	β_i_/*p*-value	β_i_/*p*-value	β_i_/*p*-value
Intercept	−0.29/.09	0.83/.00	0.49/.09	−0.71/.00	3.37/.00	1.37/.00
Gender—X_1_	0.00/.90	0.01/.67	−0.06/.02	0.02/.25	0.00/.99	−0.02/.49
Age group—X_2_	0.02/.04	−0.03/.00	0.05/.00	−0.02/.01	0.02/.22	0.03/.02
Education level—X_3_	−0.05/.08	−0.07/.02	−0.04/.25	0.01/.67	−0.02/.72	−0.05/.20
Income level—X_4_	−0.05/.00	−0.02/.07	−0.05/.00	0.02/.07	0.02/.34	0.00/.79
Work sector—X_5_	0.01/.22	0.00/.87	−0.01/.32	0.00/.97	0.02/.24	0.01/.36
Residence district—X_6_	0.00/.76	−0.01/.24	−0.02/.04	0.01/.19	0.01/.35	0.01/.22
HST—X_7_	0.19/.00	0.27/.00	−0.15/.01	0.16/.00	−0.42/.00	−0.03/.67
EQ—X_8_	−0.04/.07	0.00/.96	−0.01/.73	−0.02/.51	0.18/.01	−0.04/.41
SAT—X_9_	-	0.32/.00	0.39/.00	0.39/.00	−0.04/.66	−0.04/.57
TRU—X_10_	0.26/.00	-	0.48/.00	0.27/.00	−0.51/.00	−0.07/.25
REL—X_11_	0.27/.00	0.41/.00	-	−0.01/.84	−0.08/.32	0.30/.00
COM—X_12_	0.46/.00	0.39/.00	−0.01/.84	-	0.85/.00	0.37/.00
EXP—X_13_	−0.01/.66	−0.18/.00	−0.03/.32	0.21/.00	-	0.08/.03
IMP—X_14_	−0.02/.57	−0.04/.25	0.21/.00	0.15/.00	0.14/.03	-

^*^significant at *p* =.000; *R*^*2*^ shows the variance explained by the independent variables

*SAT* Satisfaction, *TRU* Trust, *REL* Reliability, *HST* Health Status, *COM* Comparison of the Cyprus Health System before and after the implementation of the General Health System, *EXP* Expectations, *IMP* Improvement factors, *EQ* Emotional Intelligence

Clearly, all models exhibit ANOVA F-test values corresponding to overall high statistical significance (*p* = 0.000), indicating at least one predictor significantly influences the dependent variable. Also, with the exception of EXP, all models exhibit a satisfactory or even high value for *R*^*2*^ (> 0.50).

Now, regarding the individual dependent variables, SAT seems to be influenced by Age, Education, Income, HST, EQ, TRU, REL and COM, which all exhibit *p*-values < 0.10. Similarly, TRU is influenced by Age, Education, Income, HST, SAT, REL, COM and EXP. REL is influenced by Gender, Age, Income, Residence, HST, SAT, TRU and IMP. COM is influenced by Age, Income, HST, SAT, TRU, EXP and IMP. Notably, EXP is influenced by no demographic factor, but by HST, EQ, TRU, COM and IMP, while IMP is influenced by Age only, as well as by REL, COM and EXP. Note that the intercept, representing the predicted value of the dependent variable when all predictors are 0, is statistically significant, with *p* < 0.10 for all constructs.

In particular, one can observe the following behaviors.

- Gender seems to have a negative significant influence on REL, and no significant influence on the rest of the response variables. This result actually comes as verification of the results shown in Tables 2 and 3, where females’ mean value for REL was significantly lower than that of males.
- Age influences all response variables except EXP. For SAT, REL, and IMP, the positive coefficients suggest that older individuals are more satisfied with the system, find it more reliable, and consider it more important. However, the negative coefficients for TRU and COM indicate that older individuals trust the system less and communicate less effectively compared to younger individuals. This could reflect generational differences in expectations, usage, or comparisons made when interacting with the system.
- Education level has a weak negative influence on SAT, suggesting that less educated individuals are slightly more satisfied, potentially due to less understanding of the system. Its negative influence on TRU indicates that higher education might lead to greater scrutiny or skepticism, reducing trust. The lack of significance for other variables implies that education does not directly affect perceptions of reliability, comparisons, expectations, or improvements, which may depend on other factors like personal values or exposure.
- Income level has a weak negative influence on TRU and COM, indicating that higher income individuals might imagine better access to trustworthy or communicative services should exist. Its strong negative influence on SAT and REL suggests that wealthier individuals may have higher expectations that the system fails to meet, leading to dissatisfaction and reduced reliability perceptions. The absence of influence on EXP and IMP suggests that income does not directly shape perceived expectations or the improvements of the system.
- Work sector has no influence on any response variable, a fact already indicated by the ANOVA results (Table 2), which is unexpected. This lack of impact might suggest that perceptions of the system are universal across professional groups, regardless of sector-specific expectations or needs. It also highlights the system's consistency in how it interacts with individuals, irrespective of their work sector.
- Residence district has a significant negative influence on REL, indicating that people in less populated, rural areas perceive the system as less reliable. This aligns with Fig. 2, where urban districts like Nicosia, Limassol, and Larnaca showed higher reliability ratings compared to rural districts like Paphos and Famagusta. The lack of influence on other variables suggests that perceptions of satisfaction, trust, comparisons, expectations, or improvements are more consistent across districts.
- HST strongly positively influences SAT, TRU, and COM, indicating that trust in the health system directly enhances satisfaction, trust, and communication. However, its strong negative influence on REL and EXP is intriguing, suggesting that high trust might create a complacency effect, reducing the perceived reliability and quality of expectations. Its lack of influence on IMP suggests that trust does not affect how improvements individuals find the system.
- EQ negatively influences SAT, indicating that individuals with higher emotional intelligence might be more critical and harder to satisfy. However, its positive influence on EXP shows that emotional intelligence enhances perceived quality of expectations, perhaps through better interpersonal interactions. Its lack of significant influence on other variables suggests that EQ has a focused, but limited, role in shaping perceptions.
- SAT has a strong positive influence on TRU, REL, and COM, emphasizing that satisfaction boosts trust, reliability, and communication. The lack of influence on EXP and IMP suggests that satisfaction does not directly translate to improved expectations, or perceived improvement, potentially due to differences in individual expectations and priorities.
- TRU has a strong positive influence on SAT, REL, and COM. This comes a no surprise as these constructs are strongly correlated in a positive manner (Table 1) and confirms the role of TRU in enhancing SAT, REL and COM. The negative influence on EXP indicates that higher trust might lead to fewer critical evaluations of expectations. Its lack of influence on IMP suggests that trust does not shape how improvement individuals consider the system.
- REL has a strong positive influence on SAT, TRU, and IMP. This comes a no surprise as these constructs are strongly correlated in a positive manner (Table 1) and suggests that REL enhances SAT, TRU and IMP., and perceived importance. However, its lack of influence on COM and EXP indicates that REL does not directly affect COM or EXP, confirming previous results.
- COM has strong positive influence on SAT, TRU, EXP and IMP. This comes a no surprise as these constructs are strongly correlated in a positive manner (Table 1) and underscores COM’s critical role in shaping perceptions of TRU, EXP and IMP. Its lack of influence on REL indicates that COM does not necessarily align with perceptions of REL.
- EXP has a strong positive influence on COM, showing that better expectations enhance communication. Its negative influence on TRU suggests that individuals with good expectations may trust the system less, possibly due to higher expectations being unmet in other areas. Its positive influence on IMP indicates that expectations shape perceptions of IMP.
- Finally, IMP has a strong positive influence on REL and COM, indicating that perceived IMP is tied to REL and COM. Its positive influence on EXP shows that IMP enhances EXP, suggesting that individuals who consider the system improvement are more likely to rate their EXP positively.

## Discussion

The implications of the most evident findings of the current study can be summarized as follows.

- Gender shows a statistically significant negative influence on REL, highlighting systematic gender-based differences. On the other hand, gender is not a significant predictor for other factors, suggesting minimal gender-related differences in these constructs. One may suspect that variables like age, education, or income may mediate or overshadow the influence of gender.
- Age group positively and statistically significantly affects SAT, indicating that experience, resilience, and emotional stability improve occupational satisfaction. Older people are happier because they have reasonable expectations of their roles and environments, professional security, and meaningful work and relationships. Age also positively and statistically significantly affects REL, due to life experience, socioeconomic stability, and a growing focus on relationships and communal values are exhibited by older people. Age positively affects EXP because older people have higher expectations based on their experiences and resources.
- Education level negatively influences SAT, as highly educated individuals often possess elevated expectations for roles, career progression, and organizational benefits. When unmet, these expectations, along with feelings of overqualification and heightened awareness of workplace inefficiencies, lead to reduced satisfaction. Similarly, education negatively predicts TRU, as advanced education fosters critical thinking and exposure to diverse norms, which may discourage trust.
- Income level demonstrates consistent negative relationships with SAT, TRU and REL, highlighting the complex influence of financial resources on behavioral and organizational constructs. This could suggest that higher income levels, by fostering economic stability and resource access, reduce reliance on satisfaction- and trust-based relationships. Also, individuals with higher income levels may prioritize career advancement and material success over community engagement or reliability-related behaviors.
- HST demonstrates consistent positive relationships with SAT, TRU and COM, indicating that individuals with better health status are more likely to report higher satisfaction and trust, and can be favorable in the HS comparison with the past. The negative influence of HST on REL may reflect unintended consequences such as unmet expectations or over-reliance on systems that fail to deliver as hoped. The negative influence on EXP may indicate that a very good health status can diminish further expectations.
- EQ negatively and statistically significantly affects TRU. High EQ individuals often exhibit heightened awareness of workplace dynamics, leading to lower TRU due to unmet expectations and stress sensitivity. In contrast, EQ positively predicts EXP, as motivated and engaged individuals with high EQ tend to set higher expectations.

Regarding the non-significance of some demographic variables on certain constructs, this may need further attention. This can be achieved by studying non-linear effects or interactions with other variables (for instance, Gender × Education, Gender × Income. Exploring interactions could also explain some complex behaviors. In particular, future research could investigate interaction effects, such as EQ with Age or Education, to provide a more granular understanding of these dynamics. Overall, EQ remains a critical but complex factor in shaping behaviors and outcomes, requiring balanced strategies to maximize its benefits and minimize its drawbacks.

An indirect finding that may need attention for GHS programs or interventions aiming to improve SAT should focus on enhancing TRU, REL, and COM, as indicated by their strong correlations with SAT (see Table 1). On the other hand, EXP, IMP, HST and EQ appear to operate somewhat independently of the other constructs. They may require targeted approaches rather than relying on broader interventions impacting SAT, TRU or REL.

Regarding previous studies on patients’ satisfaction and other relevant factors on MHS, accessibility and staff behavior were identified as critical drivers [24, 30], while the importance of communication and respectful treatment were also stressed [25, 34]. Also, empathetic interactions and clear explanations as key to improving patient experiences were identified [27, 31]. Operational inefficiencies, such as long waiting times and inadequate infrastructure, are recurring issues in multiple studies [24, 25, 31]. Several studies collectively emphasize the need for systemic reforms to address bottlenecks and enhance service delivery, despite differing in their contexts and focus areas [25, 27, 30, 35]. Most studies collectively emphasized the importance of integrating patient feedback into healthcare improvement strategies, and, while the studies vary in context, they converge on the significance of patient-centered care, efficient service delivery, and targeted reforms to enhance satisfaction [23, 27, 30, 31, 35].

In comparison to the studies above and other studies [21, 28, 32, 38] the present study: (i) views satisfaction primarily as a perception metric rather than a driver of economic efficiency; (ii) identifies factors such as professionalism, empathy, and communication implicitly, and not explicitly, through the constructs of trust and reliability; (iii) with a focus on demographic influences, tries to offer a robust framework for healthcare evaluation; (iv) in addition to satisfaction, emphasizes on trust and reliability, complementing the relevant findings in relation to global healthcare challenges.

## Conclusions

The current paper has investigated the General Health System (GHS) of Cyprus, focusing on public perceptions of satisfaction, trust, and reliability. The study has employed statistical methods, such as ANOVA and multiple regression, to assess interrelations among constructs such as satisfaction, expectations, and demographic variables. Key findings include low satisfaction levels nearly across all demographic groups, minimal variation in perception by gender, age, or income, and high public expectations for system improvement.

Correlation analysis underscores a strong positive relationship among SAT, TRU, REL, and COM, implying that any efforts to enhance satisfaction must also target improvements in trust, reliability, and perceived comparisons with past healthcare models. Additionally, HST positively correlates with SAT and TRU but negatively impacts perceptions of REL and EXP. Results from ANOVA indicate significant differences in SAT, TRU and REL based on age, education, and income, with older individuals and highly educated respondents being more critical of the system. Moreover, multiple regression analysis highlights that SAT is most significantly influenced by TRU, REL and COM, while income and education levels negatively impact TRU and REL. These findings suggest that targeted interventions focusing on trust-building, improving service reliability, and managing public expectations are essential for enhancing the effectiveness and public acceptance of the GHS. Furthermore, gender-based differences in REL perception warrant further investigation to address potential biases in healthcare service delivery.

All in all, supplementary to previous studies, the paper and its findings may provide actionable insights for policymakers, emphasizing the need for targeted reforms in trust-building and enhancing reliability, public confidence and overall satisfaction with the Cyprus’s GHS.

## Data Availability

No datasets were generated or analysed during the current study.

## Appendix 1

### The GHS of Cyprus

Ireland and Cyprus were the final EU countries without a universal MHS. Until 2019, Cyprus’ healthcare environment was divided into two uncoordinated sectors: the for-profit commercial sector and the severely regulated governmental sector. Whoever met socioeconomic or labor status conditions might use the nearly free public sector system. Non-eligible people could only get private, out-of-pocket (OOP) healthcare from the private or public sectors. Public healthcare system beneficiaries are undervalued because long waiting lines and limited hours forced many patients to use the private sector. Healthcare ecosystem prompted large OOP payments. OOP payments made for 57% of healthcare spending in 2018 (43% government funding). Cyprus’ total health expenditure (THE) was 6.7% of GDP, among the lowest in Europe. Healthcare’s horizontal fragmentation hindered supply- and demand-side controls such clinical and therapeutic guidelines, co-payment, medical audit programs, and pricing regulation. Inefficient capacity planning for human resources and infrastructure at both ends of the spectrum led to a glut of imaging facilities and a scarcity of general practitioners due to a lack of interaction between the two sectors. Few people participated in strategy planning, prioritizing, and resource allocation. After the 2013 financial crisis, many healthcare professionals departed the public sector due to compensation cuts and concerns about their tenured public official status, aggravating the problem. The memorandum's ban on public sector hiring until 2016 increased wait times and resource strain. Three public stakeholders dominate Cyprus'healthcare system. In 2017, the Cyprus Ministry of Health transferred public hospital operations to the State Health Services Organization as part of another memorandum-imposed restructuring. This change lets the MoH focus on regulation and avoid payer and provider duties. SHSO joined GHS like private hospitals. A Board of Directors comprising businesses, trade unions, patients, and the government administers HIO, a payer founded in 2001 as a public legal organization. Manage the GHS money, contract with all healthcare providers, enroll beneficiaries, and provide high-quality healthcare are its key objectives. After years of simmering talks among social stakeholders that nearly led to civil war and political regressions, a single-payer health insurance system was enacted on June 1, 2019. The GHS is based on a 2001 Parliament law revised in 2017. System financial issues caused most postponement. Cyprus’ GHS reform timetable is as follows: 2001/Laws were passed to reform GHS; 2003/Health insurance company founded; 2014/Multi-payer system rejected; 2015/A WHO study determined that Cyprus can only have a single-payer system; 2017/Parliament adopted the state health services organization law; 2017/The modified GHS law was unanimously ratified; 2019/Stage 1 of GHS begun; 2020/Stage 2 of GHS begun.

The GHS budget is hard-capped worldwide to ensure its long-term commercial viability. Therapeutic initiatives receive alternate funding under this compensation arrangement. Each replacement budget healing exercise generates ‘parts’ profit. The total substitute-budget amount assigned and the number of ‘parts’ eaten establish the ‘unit’ value for each temporal period of an event or entity's existence. Each activity's reimbursement amount differs, demonstrating the book acted. It helps doctors battle supplier-inferred demand and chemists run the proper number of medications. Pharmacy claw-backs help attain the budget before real strength expenditure exceeds it. Physicians cannot treat private patients below the GHS to avoid building a parallel private market and diverting beneficiaries to health management. All GHS finance contributions and copayments are shown in Table 4 in Appendix 1.

**Table 4:** Financing of the general health system of cyprus

**Contributions**	**Initial Stage**^**a**^**Salary %**	**Final Stage**^**b**^**Salary %**
Employees	1.70	2.65
Employers	1.85	2.90
Self-Employed	2.55	4.00
State	1.85	2.90
Pensioners	1.70	2.65
Income earners (rent, interest, dividends)	1.70	2.65
Public Officers	1.70	2.65
Government	1.65	4.70
**Copayment**		
Pharmaceuticals	1€ per product	
Per Laboratory tests	1€ per test or test panel	
Specialist Visit	6€ per visit	
Visit to a nurse or midwife	6€ per visit	
Healthcare service performed by a specialist doctor in radiology/diagnostic radiology	10€ per visit	
Allied healthcare provider	10€ per visit	
Emergency services	10€ per visit	
Visit to a specialist without referral from a Personal doctor	25€ per visit	

^a^2019, ^b^2020

In Cyprus’ healthcare system until 2019, profit-driven commercial and tightly controlled state sectors operated independently. Anyone who met socioeconomic or labor status criteria might use the supplemental public sector system. Not meeting these conditions meant receiving only privately funded, out-of-pocket (OOP) healthcare from the private or public sector. Public risk-sharing schemes used socioeconomic and job status to determine participation before the GHS. Non-EU nationals needed private health insurance for residency before the GHS. There was commercial health insurance. Simple insurance plans had modest maximum reimbursement rates, frequently far lower than public and commercial provider costs. Refugees, their dependents, and regular residents of Cyprus-governed territories with supplemental protection status had free treatment like Cypriot nationals before the GHS. This limited access because enrollment required a three-year unbroken social insurance fund contribution.

After careful consideration and extraordinary social acceptance, the GHS was approved in 2019. GHS efficiency measures. Hospital enrollment and public-private coordination are important challenges. The introduction of GHS changed Cyprus'history. The greatest OOP healthcare spending in Europe, fragmentation between public and private sectors, and long waiting lists necessitate a modern, patient-centered, efficient, and effective healthcare system.

## Appendix 2

### The questionnaire

Thank you for participating in this questionnaire. The purpose of this survey is to gather information about your satisfaction, trust, reliability expectations, and a comparison of your health status before and after the implementation of the General Healthcare System in Cyprus. Your responses will help us evaluate the effectiveness of the system and identify areas for improvement. Please answer the following questions to the best of your ability. Your responses will remain confidential.

**Table:** SECTION A: General information-demographics

Gender	Male	Female			
Age	18 and under	19-34	35-49	50-64	65 and above
Education	Primary School	High School	University		
Annual Income (€)	10000 and under	10001-20000	20001-30000	30001-40000	40001 and above
Work Sector	Medical/Health	Educational	Business	Services	Other
District Residence	Nicosia	Limassol	Larnaka	Paphos	Famagusta

**Table:** SECTION B: Satisfaction with the General Healthcare System (GHS)

How satisfied are you with GHS for the?	1	2	3	4	5
Access to healthcare services (availability of doctors, specialists, hospitals)					
Quality of healthcare services received					
Waiting times for appointments or treatment					
Effectiveness of preventive care services (screenings, vaccinations)					
Professionalism and competence of healthcare professional					
Information provided regarding your healthcare needs and options					
The cost-effectiveness of the healthcare services					
Personalized and patient-centered care					
Overall satisfaction with the National Health System					

Rate on a scale of 1 to 5, where 1=Very Dissatisfied, 2=Dissatisfied, 3=Neutral, 4=Satisfied and 5=Very Satisfied

**Table:** SECTION C: Trust in the General Healthcare System (GHS)

How much do you trust GHS?	1	2	3	4	5
To meet your healthcare needs effectively					
To ensure the privacy and security of your personal health information					
To have your best interests in mind					
To employ professionals that are competent and knowledgeable					
To provide timely and accurate information about your health					
To make evidence-based decisions for the overall benefit of the population					

Rate on a scale of 1 to 5, where 1=No trust, 2=Low Trust, 3=Moderate Trust, 4=High Trust and 5=Compete Trust

**Table:** SECTION D: Reliability in the General Healthcare System (GHS)

How Reliable is the GHS?	1	2	3	4	5
In providing timely access to healthcare services when needed					
In delivering accurate diagnoses and effective treatments					
In handling emergency situations efficiently					
In the competence and professionalism of healthcare providers					
In effectively addressing public health emergencies and crises					

Rate on a scale of 1 to 5, where 1=Very unreliable, 2=Unreliable, 3=Neutral, 4=Reliable and 5=Very reliable

**Table:** SECTION E: Health status

PHYSICAL HEALTH On a scale of 1 to 5, where 1= Very low, 2=Low, 3= Moderate, 4= High and 5= Very high, rate the following:	1	2	3	4	5
Your overall physical well-being					
Your engagement in regular exercise or physical activity					
Your satisfaction with your current weight and body composition					
Your sleep quality					
NUTRITION: How often in a typical week do you consume?	0	1	2	3	4+
Red meat					
Fish					
Fruits and vegetables					
Dietary supplements or vitamins					
MENTAL AND EMOTIONAL WELL BEING On a scale of 1 to 5, where 1= Almost Never, 2=Rarely, 3=Sometimes, 4=Often and 5=Almost Always, rate the following:	1	2	3	4	5
Do you generally feel stressed?					
Do you generally feel happy?					
Do you experience frequent mood swings or emotional fluctuations?					
Have you experienced any significant stressors or traumatic events recently?					
Have you been feeling consistently motivated and engaged in daily activities?					
SOCIAL and WORK WELL-BEING On a scale of 1 to 5, where 1= Very low, 2=Low, 3= Moderate, 4= High and 5= Very high, rate the following:	1	2	3	4	5
Your level of social support (family, friends, community)					
Your engagement in social activities or quality time with loved ones					
The available time for relaxation, leisure, and self-care activities.					
The level of your satisfaction with your current work					
SUBSTANCE USE AND LIFESTYLE CHOICES On a scale of 1 to 5, where 1= Never, 2=Rarely, 3=Sometimes, 4=Often and 5=Almost Always, rate the following:	1	2	3	4	5
Do you use tobacco?					
Do you consume alcohol?					
Do you use recreational drugs?					
Are there any other habits or behaviors that may impact your health negatively? (excessive screen time, lack of physical activity, irregular meal patterns)					
MEDICAL HISTORY On a scale of 1 to 5, where 1= Almost Never, 2=Rarely, 3=Sometimes, 4=Often and 5=Almost Always, rate the following:	1	2	3	4	5
How often do you have check-ups and screenings?					
Have you ever had any surgeries or medical procedures in the past?					
Have you experienced any side effects from medications in the past?

**Table:** SECTION F: Comparison before and after the implementation of GHS

	1	2	3	4	5
The impact of GHS on your ability to access necessary healthcare services					
The impact of the GHS on your overall well-being and quality of life					
Improvements in the accessibility of healthcare					
The quality of healthcare services and facilities					
The affordability of healthcare services					
The waiting times for medical appointments					
Coordination and communication among healthcare providers					
The healthcare costs are more transparent and understandable					
Did you receive more personalized and patient-centered care?					
The integration of technology in healthcare services					

Please answer the above questions by comparing the healthcare status before and after the implementation of General Healthcare System (GHS). Rate on a scale of 1 to 5, where 1=Great Negative Impact, 2=Negative Impact, 3=No significant Impact, 4=Positive Impact and 5=Great Positive impact

**Table:** SECTION G: Expectations from General Healthcare System (GHS)

	1	2	3	4	5
The primary goal of GHS is to provide universal access to healthcare services					
The primary goal of GHS is controlling healthcare costs					
The role of the government in GHS is regulating and overseeing healthcare providers					
The role of the government in GHS is funding healthcare services through taxes					
The GHS should cover primary care					
The GHS should cover emergency care					
The GHS should cover preventive care (vaccinations, screenings)					
The GHS should cover specialty care (e.g., cardiology, orthopedics)					
The GHS should cover mental health services					
The GHS should cover dental and vision care					
The monthly cost of GHS is a major concern					

On a scale of 1 to 5, where 1 = Strongly Disagree, 2 = Disagree, 3 = Neutral, 4 = Agree and 5 = Strongly Agree, rate the above statements as for the importance and the goals of the GHS

**Table:** SECTION H: General Healthcare System improvement factors

	1	2	3	4	5
Individuals should have the option to purchase private health insurance in addition to the GHS					
Individuals should have the option to choose between private health insurance and GHS					
The access to healthcare Services is sufficient (primary care, specialized medical services)					
The monthly cost (contribution rate based on income) for individuals is high					
The quality of care is high (timely and appropriate treatment, healthcare professionals are well-trained and skilled)					
The level of health education and promotion is high (initiatives to promote healthy lifestyles, effective awareness programs, prevention and early intervention strategies)					
The level of healthcare infrastructure is high (modern and up-to-date, facilities are well-maintained and adequately equipped, sufficient number of healthcare facilities)					
The level of the healthcare workforce is high (adequate number of healthcare professionals, healthcare workforce is well-distributed across regions, programs to continuously train and develop healthcare professionals)					
The level of healthcare financing is high (affordability of healthcare services)					
The level of health information system is high (health records and information, Information is securely managed and protected)					
The level of patient satisfaction and feedback is high (there are channels for patients to provide feedback and suggestions, feedback is actively used for improving services)					
The level of Government policies and regulations are high (effective regulation of healthcare providers and services, policies are flexible and adaptive to changing healthcare needs)					
The level of emergency response and preparedness is high (well-prepared for emergencies, response times during emergencies are satisfactory, emergency services are well-coordinated)					

Rate on a scale of 1 to 5, where 1=Strongly Disagree, 2= Disagree, 3=Neutral, 4=Agree, 5= Strongly Agree

**Table:** SECTION I: Emotional intelligence questionnaire

	1	2	3	4	5
I am aware of my own emotions and can accurately identify them					
I am good at recognizing emotions in others based on their facial expressions and body language					
I am able to regulate and manage my own emotions effectively					
I can empathize with the feelings and perspectives of others					
I am skilled at resolving conflicts and disagreements in a constructive manner					
I am able to motivate and inspire others					
I am generally optimistic and have a positive outlook on life					
I am able to adapt to changes and handle stressful situations effectively					
I am good at communicating my emotions and needs to others.					
I am able to set and achieve meaningful goals for myself					

Please read each statement carefully and indicate how often each statement applies to you. Choose the response that best represents your typical behavior. Rate on a scale of 1 to 5, where 1= Almost Never, 2=rarely, 3=Sometimes, 4=Often and 5=Almost Always
